# Cyclodextrins in Antiviral Therapeutics and Vaccines

**DOI:** 10.3390/pharmaceutics13030409

**Published:** 2021-03-19

**Authors:** Susana Santos Braga, Jéssica S. Barbosa, Nádia E. Santos, Firas El-Saleh, Filipe A. Almeida Paz

**Affiliations:** 1 LAQV-REQUIMTE, Department of Chemistry, University of Aveiro, 3810-193 Aveiro, Portugal; jessicambarbosa@ua.pt (J.S.B.); nadiaasantos@ua.pt (N.E.S.); 2 CICECO—Aveiro Institute of Materials, Department of Chemistry, University of Aveiro, 3810-193 Aveiro, Portugal; filipe.paz@ua.pt; 3 Ashland Specialty Ingredients, Paul-Thomas Strasse, 56, D-40599 Düsseldorf, Germany; FElSaleh@ashland.com

**Keywords:** antiviral drugs, inclusion complexes, cyclodextrins, vaccines

## Abstract

The present review describes the various roles of cyclodextrins (CDs) in vaccines against viruses and in antiviral therapeutics. The first section describes the most commonly studied application of cyclodextrins—solubilisation and stabilisation of antiviral drugs; some examples also refer to their beneficial taste-masking activity. The second part of the review describes the role of cyclodextrins in antiviral vaccine development and stabilisation, where they are employed as adjuvants and cryopreserving agents. In addition, cyclodextrin-based polymers as delivery systems for mRNA are currently under development. Lastly, the use of cyclodextrins as pharmaceutical active ingredients for the treatment of viral infections is explored. This new field of application is still taking its first steps. Nevertheless, promising results from the use of cyclodextrins as agents to treat other pathologies are encouraging. We present potential applications of the results reported in the literature and highlight the products that are already available on the market.

## 1. Introduction

Viral infections pose strong risks to human health and survival [1]. In 2017, diseases caused by virus agents were responsible for more than 2% of the global death count of 56 million, with infection from human immunodeficiency virus (HIV) causing more than 954,000 casualties (c.a. 1.7%), and hepatitis (A, B, and C) and influenza infections accounting each roughly 125,000 deaths (c.a. 0.2%) [2]. Recent outbreaks of viral infections further contribute to demonstrate that, in spite of the efforts made by health care organisations around the globe to reduce and control the impact of these pathogens on human health, threats may arise without warning and can easily spread. Examples include the severe acute respiratory syndrome (SARS) caused by a novel coronavirus (CoV) designated SARS-CoV that broke out in China from 2002 to 2003 [3], the pandemic influenza caused by a swine H1N1 influenza A virus in 2009 [4], the Middle East respiratory syndrome (MERS) in 2012 caused by a new virus called MERS-CoV [5], the Ebola outbreak in West Africa between 2014 and 2016 [6], and the currently ongoing outbreak of a coronavirus infectious disease (COVID-19) that started in the last months of 2019 and is attributed to a new virus, SARS-CoV2. The complex evolution of the current pandemic has been pushing its resolution to an increasingly distant horizon. Three new and more infective variants emerged at the end of 2020, some of them potentially more virulent. Vaccination, the eagerly anticipated solution, is still taking its first steps and progressing slowly, with much yet to be learned about efficacy towards new variants and the actual length of immunisation [7].

In the future, more outbreaks can be expected as the increased pressure on once untouched ecosystems will put mankind in close contact with unknown mammal viruses, estimated to exist in high numbers as much as 320,000, and to have a strong possibility of transmission to humans [8]. Tackling these emerging viral infections requires strong research efforts with a multidisciplinary scope that integrates simple approaches, such as repurposing drugs [9], and in-depth, long-term studies to develop new active chemical entities [10], medicinal biomolecules [11], and vaccines [10]. Moreover, actives often require the support of adequate inert materials to ensure stabilisation, solubilisation [12], and bioavailability [13,14].

**Figure 1:**
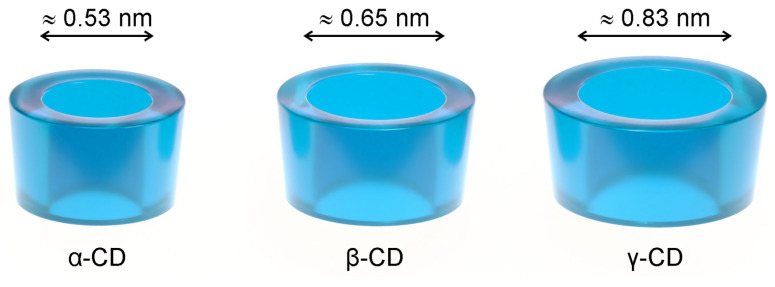
The three most abundant native cyclodextrins (CDs), α-CD, β-CD, and γ-CD, schematically drawn as truncated cones. An estimate of the inner cavity diameter is presented for each [20].

Cyclodextrins (CDs) are used as solubilisers, taste-masking, and stabilising agents in various drug formulations, from solid oral dosage forms to injectables [15,16], also appearing as ingredients in cosmetics [17,18] and food products. Native cyclodextrins are naturally occurring cyclic oligosaccharides composed of six to eight α-1,4-linked D-glucose units (α-, β- and γ-CD). They have the shape of a truncated cone (Figure 1), with the secondary hydroxyl groups facing the wider rim and the primary ones facing the narrower rim. This unique geometry allows them to dissolve fairly well in water while keeping a hydrophobic cavity that accommodates molecules with a size and geometry adequate to each cyclodextrin. While the solubilising effect is the most obvious consequence of the geometry of cyclodextrins, these hosts are able to mask the unpleasant taste of included guest molecules and protect them against oxidation and the damaging action of heat or UV radiation [19,20,21].

Native cyclodextrins are quite safe for ingestion because they are practically not absorbed from the gastrointestinal (GI) tract. In Japan, they are even considered natural products and their use in foods is very widespread. In the rest of the world, they are GRAS products, i.e., they are ‘generally regarded as safe’ to ingest from foods [22,23,24] and oral pharmaceutical dosage forms [16,25,26,27]. Two of the native cyclodextrins (α-CD and β-CD) should not, however, be administered directly into the bloodstream, because they have renal toxicity [16]. Moreover, native cyclodextrins are haemolytical, (observed in vitro at concentrations of 6, 3, and 16 mM for α-, β- and γ-CDs, respectively) [28] as a result of their ability to extract phospholipids and cholesterol from the erythrocyte membrane [29].

**Figure 2:**
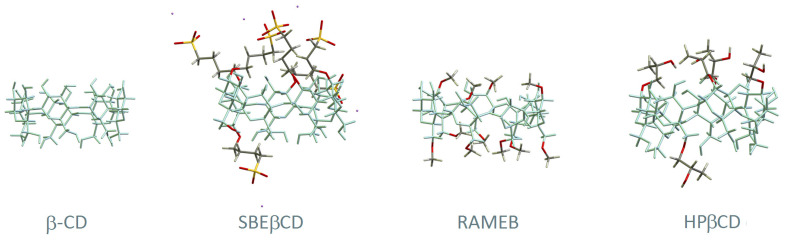
Structural representation of β-CD and three of its derivatives: (2-hydroxy)propyl-beta-cyclodextrin (HPβCD), randomly methylated beta-cyclodextrin (RAMEB), and sulfobutyl ether β-CD (SBEβCD). The main skeleton of β-CD is represented in blue and the substituent groups are highlighted with different colours (carbon in grey, oxygen in red, hydrogen in white, sulphur in yellow and sodium in purple).

Chemically modified cyclodextrins are obtained from the native ones through functionalisation of their hydroxyl groups with a myriad of substituents and combinations thereof. A report of 2012 mentioned more than 1500 known cyclodextrin derivatives [30], with the current number expected to be much higher. The most used ones in the field of pharmaceutics are represented in Figure 2. 2-hydroxypropylated (HP) derivatives are among the ones with the most widespread use, in particular HPβCD for its high safety and tolerability. HPγCD is restricted to topical use at a maximum concentration of 1.5% (*w*/*v*). Methylated derivatives are also employed in a few pharmaceuticals, particularly in RAMEB and DIMEB. DIMEB, or heptakis-2,6-di-O-methyl-β-CD, is a per-functionalised derivative at the hydroxyl groups 2 and 6. It has moderate hepatic toxicity, with doses of 300 mg/kg in mice causing increased levels of two biomarkers of hepatic injury: glutamate–pyruvate transaminase and glutamate–oxaloacetate transaminase [31]. Nevertheless, trace amounts of DIMEB are found in three injectable bacterial vaccines [32], in which DIMEB was used as a culture medium modifier for growing the bacteria. RAMEB is a randomly methylated beta-cyclodextrin averaging 1.8 methoxyl groups per glucose unit. It can only be used in topical formulations because of its high affinity to cholesterol which results in strong haemolytic activity [33,34] and renal toxicity superior to that of the parent β-CD. Another biocompatible CD is sulfobutyl ether β-CD (SBEβCD), developed to be non-nephrotoxic and present in several FDA-approved pharmaceuticals for both oral and intravenous administration [35]. Table 1 presents a summary of pharmaceutical uses of cyclodextrins, along with their toxicity and restrictions to use, compiled from data of European Medicines Agency (EMA) [16], the United States Food and Drugs Administration (FDA) [25,26,27,32,35], and the joint WHO/FAO Expert Committee on Food Additives (JECFA) [22,23,24].

Information in this table was compiled from the European Medicines Agency (EMA) [16], the United States Food and Drugs Administration (FDA) [25,26,27,32,35], and the joint WHO/FAO Expert Committee on Food Additives (JECFA) for the oral intake [22,23,24].

## 2. Cyclodextrins and Antiviral Drugs

**Figure 3:**
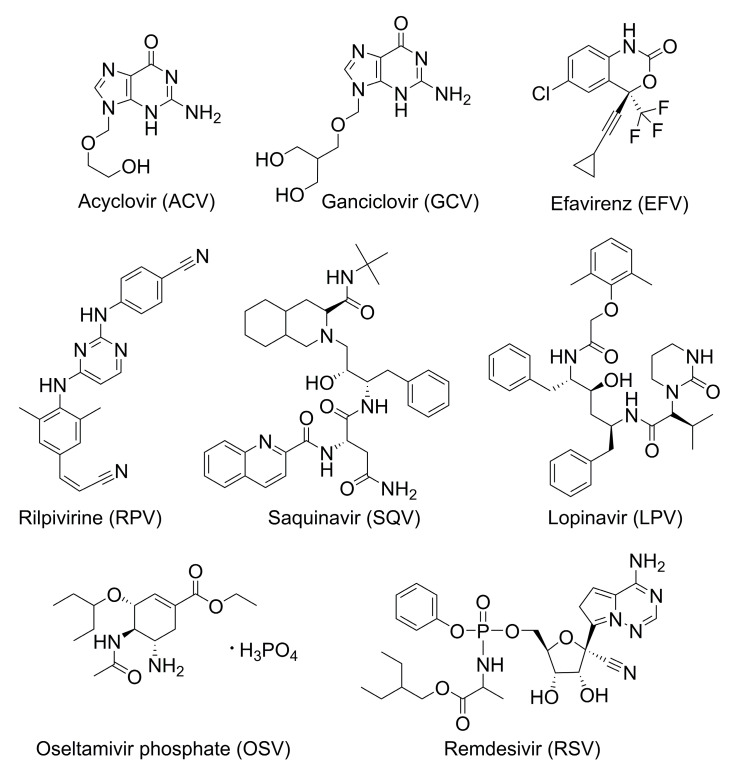
Commercially available antiviral active pharmaceutical ingredients (APIs) reported to form inclusion complexes and/or interaction products with native and chemically modified cyclodextrins.

Cyclodextrins interact with various active pharmaceutical ingredients (APIs) used in the treatment of viral diseases (Figure 3), either through the formation of inclusion compounds or when employed in excess amounts to obtain combined products with increased solubility and/or activity.

### 2.1. Acyclovir

Acyclovir (ACV), or 9-((2-hydroxyethoxy)-methyl)-guanine, is a synthetic guanine analogue active against several species of the *herpesviridae* family, in particular against *Human alfaherpesvirus* types I and II, commonly named as the herpes simplex virus (HSV), and also the type III, which is commonly known as the varicella-zoster virus (VZV). Acyclovir is usually administered in the form of tablets, with dosage strengths of 200 mg taken four times daily. Other available dosage strengths are 400 mg and 800 mg (the latter only available in the USA) [36]. Topical dosage forms such as gels and lotions are also available as non-prescription relief for small dermal eruptions associated with HSV [37]. Acyclovir is slightly soluble in water, having a log *p*-value of −1.8 and featuring solubility values in the interval of 1.2–1.6 mg/mL at 25 °C. Solubility increases with temperature to reach 2.5 mg/mL at 37 °C [38]. Acyclovir doses of 200 and 400 mg can be tentatively classified as belonging to the biopharmaceutical classification system (BCS) class III [39], while the 800 mg dose has a more irregular absorption profile and, for this reason, it is not classified [38].

Several studies on the interaction of acyclovir with cyclodextrins are reported, most of them attempting to ameliorate solubility and bioavailability. Inclusion of acyclovir into β-CD in an equimolar ratio only affords a moderate solubility increase of c.a. 1.9-fold (from 27% with pure acyclovir to roughly 47% with the complex), and without much change to the dissolution profile of the drug, mainly because the drug itself already dissolves quickly reaching a plateau within less than 10 min [40,41]. Noteworthy, increasing the amount of β-CD in the inclusion complex also does not necessarily lead to increased solubility. This was reported for the kneaded 5:1 (β-CD: ACV) adduct that had, in the best-case scenario, a solubility increase of c.a. 1.5-fold in regard to the pure drug [42]. The influence of β-CD inclusion on the pharmacokinetic profile of acyclovir was, thus, evaluated using the β-CD·ACV complex with 1:1 stoichiometry, with a dose equivalent to 75 mg/kg given to Sprague–Dawley male rats in the form of a suspension by direct insertion into the intestine. Results showed a slight reduction in the plasma peak (C_max_), from 7.82 μg/mL for pure acyclovir to 5.22 μg/mL for the complex. The time needed to reach the peak (*t*_max_) was also longer for β-CD·ACV, 60 min, while pure acyclovir took only 40 min. A possible explanation is that acyclovir absorption implies its release from the complex, and this seemingly occurred in a sustained fashion when the drug was included in β-CD. Nevertheless, the total dose of the drug (AUC_total_) found in the plasma of rats was higher by roughly 1.2-fold, that is, AUC_total_ of β-CD·ACV was 2119 μg·min/mL, whereas for pure acyclovir it was 1748 μg·min/mL [43].

Acyclovir inclusion into HPβCD was reported, both for the 5:1 ratio [42] and the 1:1 ratio [44]. The 5:1 product with HPβCD improved the solubility of acyclovir in a neutral aqueous medium by c.a. 1.5-fold [42]. The 1:1 HPβCD·ACV adduct, prepared by kneading, was able to increase solubility in an acidic medium (HCl 0.1N) to 100%. Pure acyclovir, in the same medium, dissolved up to 80%. Regarding bioavailability, Wistar male rats taking an oral dose of HPβCD·ACV that corresponded to 20 mg/kg of pure acyclovir exhibited a plasma peak (C_max_) value averaging 3.37 ± 0.75 μg/mL, a *t*_max_ of 45 min, and an AUC_0−α_ of 483.68 ± 52.82 μg·h/mL (at which AUC_0−α_ expresses accumulated drug in the plasma during the observation time) [44]. Bioavailability of included acyclovir, measured by the AUC_0-α_, increased by 1.6-fold in regard to the control (control consisted of an equivalent dose of acyclovir and HPβCD in the form of a physical mixture). A comparison between the effects of the two cyclodextrins, β-CD and HPβCD, on the bioavailability of acyclovir cannot, however, be made because the studies were conducted with different dosages (a linear dose/bioavailability response may not occur), different controls, and slightly different routes of administration: HPβCD·ACV was administered per orum, while βCD·ACV was given directly at the intestine, thus excluding possible contributions from absorption at the stomach and initial segments of the duodenum.

Loading of acyclovir into cyclodextrin-conjugated polymers such as β-CD-poly(amidoamine), or β-CD-PAA [45], and β-CD-hyaluronic acid [46] was reported. These conjugated polymers can bind to several molecules of cyclodextrins, which provides them with a high loading ability by retaining a guest drug through inclusion complexes and non-inclusion interactions. The release rate of acyclovir from the β-CD-PAA carrier was evaluated in aqueous solutions with PBS and pH values of 5.5 and 7.4, both showing relatively slow release rates. At pH 7.4, only 30% of the drug had been released after 60 min; after 120 min, the release rate reached 48%. At the acidic pH of 5.5, the drug is protonated and it is, thus, more soluble. For this reason, higher amounts were released, that is, 40% after 60 min and 60% after 120 min. The acyclovir-loaded β-CD-PAA polymer was further evaluated regarding antiviral activity, by testing its growth inhibition action on kidney fibroblast cells (Vero cell line) infected with two strains of the herpes simplex virus, HSV-1 BGM, and HSV-1 MRC. At 72 h of incubation, acyclovir-loaded β-CD-PAA had EC_50_ values roughly six times lower than those of pure acyclovir, which was ascribed to the facilitated membrane entry of the polymer via pinocytosis [45]. Application prospects for the polymer remain, however, unclear. If, on the one hand, dissolution studies point to oral administration by evaluating the performance at pH 5.5, the average pH stomach when containing food; on the other hand, the ability of the polymer carrier to enter the organism by enteric absorption was not looked into. Further studies are required to elucidate the true value of β-CD-PAA as a carrier for acyclovir and other antiviral drugs.

The β-CD-hyaluronic acid conjugated polymer was shown to work as a sustained release system for acyclovir, with shortly over 10% drug release after 24 h immersion into a pH 7.4 PBS solution, roughly 45% release at 72 h and 80% release being reached only after 240 h (10 days). Growth inhibition against HSV-1 in Vero cells was evaluated at 72 h of incubation, with the polymer carrying acyclovir showing lower activity than the pure drug [46], which is an expectable result based on the quite slow drug release profile (less than half the dose is available at 72 h).

### 2.2. Ganciclovir

Ganciclovir (GCV), or 9-[1,3(dihydroxy)-2-(propoxy)-methyl]guanine, is a nucleoside analogue active against *herpesviridae* such as the Epstein–Barr virus, the herpes simplex virus (HSV), the varicella-zoster virus (VZV), and the *Human betaherpesvirus 5*, commonly referred to as human cytomegalovirus (HCMV). The latter is a typically opportunistic virus that infects and causes retinitis in immunocompromised hosts such as organ recipients or HIV-infected patients. Ganciclovir has the particularity of being one of the few antiviral drugs available to treat HCMV-associated retinitis. It is sparingly soluble in water (2.6 mg/mL), having a log *p*-value of 0.022 [47,48]. The sodium salt form of ganciclovir, commonly used in injectable formulations, has higher solubility, 7.95 mg/mL, and a log *p*-value of –1.8 [49]. Treatment of cytomegalovirus infections with ganciclovir usually involves intravenous perfusion of 5 mg/kg twice daily for 14–21 days. Ganciclovir presents hematologic toxicity, which may create the need for a dose reduction to better meet the profile and response of the patient. These adjustments limit the effectiveness of the treatment [50].

Cyclodextrin inclusion as a means to increase the activity of ganciclovir was investigated in two studies. The first report attempted inclusion into the three native cyclodextrins by the co-dissolution method at a starting proportion of 1:1. After evaporation and freeze-drying, only one of the three isolated solid products was identified as a true inclusion compound—β-CD·GCV [51]. For the evaluation of the effect of cyclodextrins on the antiviral activity of ganciclovir, another set of adducts was prepared using a host-to-guest ratio of 10:1, i.e., a quite large excess of cyclodextrin. The antiviral activity tests were conducted on fibroblasts of the MRC5 cell line infected with two different viral strains: AD169, a reference sensitive strain, and RCl1, a drug-resistant strain. Both β-CD and γ-CD were able to increase the in vitro antiviral activity of ganciclovir measured by the plaque reduction assay method, with IC_50_ against the AD169 strain having values of 1.20 ± 0.10 μM for ganciclovir, (note that low level resistance is defined as 8 μM ≤ GCV IC_50_ ≤ 30 μM), 0.70 ± 0.05 μM for β-CC:GCV (10:1) and 4.12 ± 0.48 γ-CD (10:1) [51]. Antiviral activity was also measured by the enzyme-linked immunosorbent assay (ELISA) method, with the results being summarised in the two first lines of Table 2.

In a follow-up study, the 10:1 product of β-CD with ganciclovir was selected for evaluation of the activity against clinical isolates of the virus, obtained from the trachea of a bone marrow transplanted patient (strain 2288), from the urine of renal transplanted patients (strains 539 and 731) and from a child with a congenital infection (strain 1558). The antiviral activity was increased by the presence of β-CD for all of the strains except for the 731 isolates, with the most significant increase in the activity being 24-fold against the 2288 isolate (Table 2) [52]. While these results are academically interesting because they clearly demonstrate that lower doses of ganciclovir can be used by combining this drug with cyclodextrins, we must highlight that the tested administration route (intravenous) rules out clinical applications in humans because neither β-CD nor γ-CD is currently approved for parenteral use. It is, thus, recommended to re-evaluate the activity of ganciclovir under the solubilising effect of adequate β-CD derivatives, such as HPβCD or SBECD.

HPβCD was, thus far, only investigated as a carrier for the local delivery of ganciclovir prodrugs to the retina. Results have shown that the presence of 5% HPβCD increased the in vitro corneal permeation by 2.56 fold in the case of a solution containing the dibutyrate diester prodrug of ganciclovir. Permeation of other acyl esters prodrugs was also increased but to a lower extent [53]. We note that acyl esters of ganciclovir are experimental compounds; currently, the only approved prodrug of ganciclovir is its *L*-valyl ester, valganciclovir.

### 2.3. Efavirenz

Efavirenz (EFV), or (S)-6-chloro-4-(cyclopropylethynyl)-1,4-dihydro-4-(trifluoro- methyl)-2*H*-3,1-benzoxazin-2-one, is a non-nucleoside reverse transcriptase inhibitor used as a first-line treatment for HIV-1 infection [54]. This compound is practically insoluble in water, having a solubility of c.a. 3.0–9.0 µg/mL and a log *p* of 2.07 ± 0.12 [55]. Its physicochemical properties translate into low bioavailability (40–45%) and a considerably low intrinsic dissolution rate, which will inherently interfere with the absorption and further therapeutic action [56,57].

**Figure 4:**
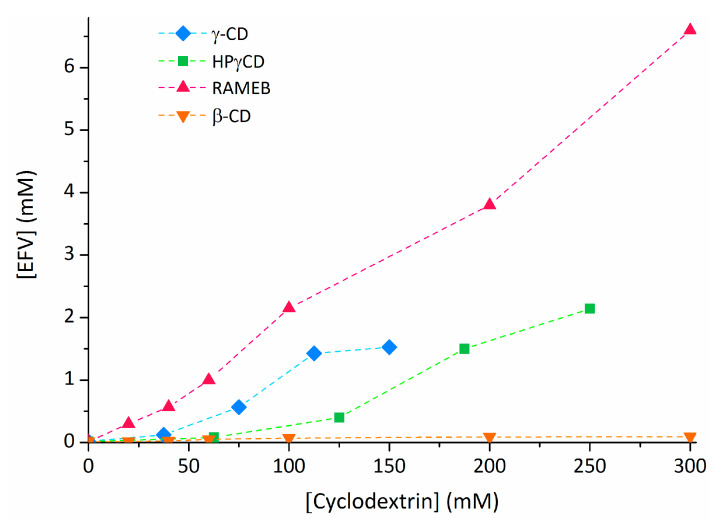
Comparison of the solubility isotherms of efavirenz (EFV) with different cyclodextrins. Adapted from literature reports on EFV with β-CD and RAMEB [56]; γ-CD and HPγCD [54].

The aqueous solubility of efavirenz can be improved by interaction with a variety of cyclodextrins. This is demonstrated by the solubility isotherms of efavirenz with β-CD, γ-CD, HPβCD, HPγCD, and RAMEB (Figure 4) [54,56].

Within these hosts, RAMEB appears to be the most effective solubiliser for efavirenz, followed by γ-CD (albeit this host showed a solubilisation plateau above 112.5 mM). β-CD was the least effective, having a very small effect on the solubility of efavirenz.

The literature presents conflicting results on the effect of HPβCD on the solubility of efavirenz. One study reported that 60 mM of HPβCD was sufficient to increase EFV solubility to roughly 1 mM [56], while another showed it to have a lower solubilising action than γ-CD, with a concentration of 125 mM of HPβCD being required to solubilise c.a. 0.5 mM of EFV [54].

In the solid state, the interaction was studied with both β-cyclodextrins (including HPβCD and RAMEB) and γ-cyclodextrins, which have a wider cavity. Various methodologies were employed to attempt inclusion. In a study comparing the inclusion abilities of two native hosts (β-CD and γ-CD) by co-precipitation, β-CD was deemed unable to include efavirenz. These conclusions were reached through the combined observation of the co-precipitate under the optical microscope, showing that the two components precipitated separately, in addition to the observation of the melting thermal transition of efavirenz in the DSC trace of the co-precipitate [54]. Another study compared kneading with co-dissolution/freeze-drying procedures, starting from a mixture of isopropyl alcohol and water (3:4) to dissolve the guest, and β-CD and ethanol when HPβCD and RAMEB were used as hosts. The products of the two methodologies were mainly amorphous, although traces of crystalline efavirenz were still visible in the powder diffractograms of the two products with β-CD and the kneaded mixture with HPβCD [56]. This shows that inclusion was not fully successful. The dissolution profiles of the products in ultrapure water under sink conditions showed that, while pure efavirenz dissolved only c.a. 10% after 180 min, the products (in the equivalent amount of drug) dissolved four- to six-folds more efavirenz. The best dissolution performance was observed for the freeze-dried product with HPβCD, which reached maximum dissolution of the drug at 50 min of immersion and maintained it through the end of the assay (180 min). Another study confirmed that γ-CD is the adequate host for efavirenz due to its wide cavity diameter. The preferential stoichiometry of inclusion was first studied in solution by ^1^H NMR to reveal the occurrence of a 3:2 (host-to-guest) complex. Then, γ-CD and efavirenz were co-dissolved in these proportions and freeze-dried to obtain (γ-CD)_3_·(EFV)_2_ as a quasi-amorphous solid product, which, after rehydration to restore its structure, proved to be isotypical with previously known γ-CD complexes, i.e., it features cyclodextrin units packed as channels with the guest molecules housed inside [58].

Pre-formulation studies seeking to develop liquid products with efavirenz resorted to interaction with two β-CD-derived polymers, a β-CD-homopolymer and a β-CD-lactose-copolymer (both linked by condensation with cyanuric chloride) [57]. The copolymer loaded with efavirenz showed an aqueous dissolution rate of the drug very close to 95% at 60 min, while for the β-CD-homopolymer the dissolution rate was roughly 85%.

Ternary systems are another alternative for ameliorating efavirenz solubility. Kneaded mixtures of efavirenz with RAMEB and low amounts of polyvinylpyrrolidone (PVP) K-30 (c.a. 1% of the total mass of the 1:1 efavirenz–RAMEB adduct) afforded a drug dissolution rate of 80% after 30 min in a 0.5% sodium lauryl sulphate (SLS) solution. For comparison, pure efavirenz dissolved 20% in the same time and conditions [59]. Ternary systems of efavirenz with β-CD and PVP K-30 are suitable for use in oral solid dosage forms: tablets obtained by direct compression showed good dispersibility and a higher dissolution rate than tablets obtained with efavirenz alone [60].

### 2.4. Rilpivirine

Rilpirivine (RPV) is a second-generation non-nucleoside reverse transcriptase inhibitor used in the treatment of HIV infection. The second generation of drugs is designed for higher potency, longer half-life, and lower incidence of adverse effects than in first-generation drugs (e.g., efavirenz). With an aqueous solubility of 0.0166 mg/mL [61] and a log *p*-value of 4.86 [62], rilpivirine is a BCS class II drug (low solubility and high permeability). Its absorption and bioavailability will thus be limited by the amount of dissolved drug in the GI tract.

The elongated shape of rilpivirine makes it adequate for inclusion into β-CD and its derivatives, with positive results on solubility and dissolution. Kneaded and co-evaporated solid products of rilpivirine with β-CD and HPβCD (host-to-guest ratio of 2:1) showed improved dissolution profiles, with more than 95% of RPV dissolved from the (HPβCD)_2_:RPV co-evaporated product after 120 min in a 0.01N HCl aqueous solution at 37 °C and approximately 90% dissolution for the remaining products. For comparison, the pure drug after 120 min under the same conditions dissolved only 25% [63]. Loading of rilpivirine into cyclodextrin-based nanosponges is also reported. One study employed diphenylcarbonate cross-linked β-CD nanosponges to load rilpivirine and increase its dissolution profile from 35% to 85% (after 40 min in 0.1 M HCl aqueous solution at 37 °C) [64]. The pharmacokinetics of the RPV-loaded β-CD nanosponges was further evaluated using Sprague–Dawley rats to show an increase in the plasma peak by approximately two-folds and a half-life increase from 4 h (pure drug) to 7 h. In another study, different types of linkers for the β-CD nanosponges were tested [61]. The resulting nanosponges were loaded with rilpivirine and their oral bioavailability was also roughly two-fold higher than that of the pure drug (as is had been observed in the previous study). This is evidence that the bioavailability increase is dependent on the ability of β-CD to release and help the drug solubilise in vivo, and not so much on the chemical composition of the nanosponge itself.

### 2.5. Saquinavir

Saquinavir (SQV) was the first HIV protease inhibitor to be approved by the FDA, in 1995. It remains in clinical use until today because of its excellent safety profile. This non-hydrolysable peptidomimetic molecule inhibits the HIV-1 protease enzyme that is responsible for cleaving polyproteins, thus interfering with the structural integrity of viral particles. Saquinavir is a weak base, its neutral form having a log *p* of 1.9 [65] and an aqueous solubility of 207 ± 5 μg/mL at neutral pH [66]; solubility values increase as the pH lowers to 5 [67]. Saquinavir is also available as a mesylate salt, for which different values of solubility were reported, from 0.293 ± 0.007 mg/mL [68] to 2.1 ± 0.3 mg/mL [66]. Saquinavir has an exceptionally poor oral bioavailability (approximately 4%) that limits its efficacy. For this reason, the current therapeutic indication involves co-administration of ritonavir, a potent enzyme inhibitor that increases the bioavailability, serum concentration and overall efficacy of saquinavir.

The reported strategies of inclusion complexation for bioavailability improvement refer to HPβCD and RAMEB. The first report on saquinavir inclusion into HPβCD was from the research group of Duchéne and Ponchel and it dates back to 2001 [69]. Successful inclusion was achieved by co-dissolving HPβCD with an excess of saquinavir, filtering the solution to isolate the dissolved complex followed by freeze-drying. The solubilising effect of HPβCD on saquinavir, with a two-fold increase, was demonstrated in another study [70]. Saquinavir was reported to interact with hydroxybutenyl-β-cyclodextrin (HBenβCD), a hydroxyalkenyl derivative of β-CD. This is still an experimental CD derivative as its toxicological safety profile has not yet been determined. Studies on the influence of HBenβCD on the dissolution of saquinavir base and saquinavir mesylate in buffer at pH 6.8 showed dissolution improvement for both, from values of 16.2% and 4.4%, respectively, for their pure forms, to values of c.a. 95% and 90% for the corresponding adducts. These values were reached after 30 min of the dissolution assay and remained constant for 6 h [66].

Adducts of RAMEB with saquinavir (base) and saquinavir mesylate are reported to help ameliorate the drug’s bioavailability. Adducts were prepared in the solid state with both saquinavir (base) and saquinavir mesylate, having displayed different ratios of drug inclusion in the final products that correspond to host-to-guest proportions (H:G) of 3:1 and 3:2, respectively [69]. Bioavailability studies were conducted using solutions of saquinavir in potassium buffer at pH 7.2 and added with RAMEB (at c.a. 2:1, H:G) administered to rats by gavage into the stomach. The presence of RAMEB afforded a more stable and rapid oral absorption, peaking at T*_max_* = 30 min and reaching a maximum plasma level, C*_max_*, of 1347.88 ± 276.76 ng/mL (from a starting dose of 20 mg/mL); the absolute bioavailability was 16.34 ± 1.12%. For comparison, the same dose of saquinavir without RAMEB addition had T*_max_* = 384 min, C*_max_* = 117.24 ± 35.77 ng/mL and an absolute bioavailability of 3.11 ± 1.14% [69].

### 2.6. Lopinavir

Lopinavir is a potent inhibitor of HIV-1 protease used in the treatment of HIV-1 infections in paediatric and adult patients and, more recently, as an off-label treatment for infection with SARS-CoV-2, albeit with no clinical benefits were observed [71], and the risk of cardiac adverse effects were raised by 14% [72]. Lopinavir is a BCS class IV drug, i.e., it has low permeability, with a log *p* of 4.7, and very low solubility in aqueous media, namely, 2.27 μg/mL in pure water [73] and 2.93 ± 0.08 μg/mL in PBS at pH 7.0 [74]. In addition to the low solubility and permeability, which hinder its absorption in the GI tract, lopinavir bioavailability is further reduced by pre- and post-systemic metabolisation involving enzymes of the cytochrome P-450 complex and their analogues. For this reason, lopinavir is currently available as a co-formulation with sub-therapeutic amounts of ritonavir, which serves to inhibit P-450 metabolism and thus ensure adequate bioavailability. However, the presence of ritonavir may bring gastrointestinal complications and metabolic abnormalities (hyperlipidaemia, glucose intolerance) [75]. A formulation in which lopinavir is better solubilised and, therefore, more available for absorption would allow reducing its time of permanence in the intestinal lumen and its intestinal metabolisation, thus posing an excellent alternative to the currently available combined therapy.

Cyclodextrins were shown to be good solubilising agents for lopinavir. One study used solubility isotherms to determine the ability of each cyclodextrin to increase lopinavir solubility, which followed the order γ-CD > HPβCD > RAMEB. Dissolution rates of lopinavir from kneaded mixtures with these cyclodextrins showed slightly different results, with RAMEB and HPβCD products dissolving quickly in the first 30 min and then reaching plateaus of c.a. 30% and 50% after 120 min, while the kneaded product with γ-CD presented a gradual dissolution over 90 min to reach a final dissolved rate of c.a. 55% after 120 min [73]. Another study used γ-CD, commercial HPγCD and a new form of HPγCD having a high degree of substitution, (HP)_17_γCD (DS = 17.1 ± 1) and evaluated the solubility after 24 h in PBS at 37 °C using a single point test (at unspecified CD concentrations and also with excess lopinavir); solubility values were increased by 87-, 114-, and 129-folds, respectively, in comparison with pure lopinavir [76]. Solid inclusion complexes with γ-CD, HPγCD, and (HP)_17_γCD were prepared by two procedures, i.e., co-evaporation and spray drying. The spray-drying method was more adequate for obtaining co-amorphous products, while the co-evaporated 1:1 products still showed some crystallinity; spray-dried products were also better at increasing lopinavir dissolution rates.

### 2.7. Oseltamivir

Oseltamivir phosphate (OSV), popularly known under its brand name, Tamiflu^TM^, is a neuraminidase inhibitor active against influenza A and B strains. It is a fairly polar drug, with a log *p*-value of 0.36 [77] and aqueous solubility of 1.6 mg/mL [78].

OSV inclusion addressed no solubility issues, aiming rather at masking its bitter taste. A solid product with OSV and β-CD, prepared by co-stirring the two components and freeze-drying the mixed solution, was evaluated by a panel of six non-trained tasters, which rated its bitterness at a value of 2. This value was lower than that of pure oseltamivir, which was rated 4 (scale from 0 to 4) [79]. The use of β-CD, HPβCD, RAMEB, DIMEB, and the sulfomethyl-ether and maltosylated derivatives is patented in China for the production of improved solid and liquid oral OSV formulations [80].

### 2.8. Remdesivir

Remdesivir (RSV) is an investigational drug with a broad antiviral spectrum of activity. Previously studied in Ebola and MERS outbreaks, remdesivir started to be tested against SARS-CoV-2 as soon as the pandemic began, having shown positive results in a few clinical studies [81,82]. In April 2020, remdesivir was approved by the EMA for compassionate use in patients with severe cases of SARS-CoV-2 infection, i.e., those requiring invasive mechanical ventilation [83], with the FDA following in May [84].

**Figure 5:**
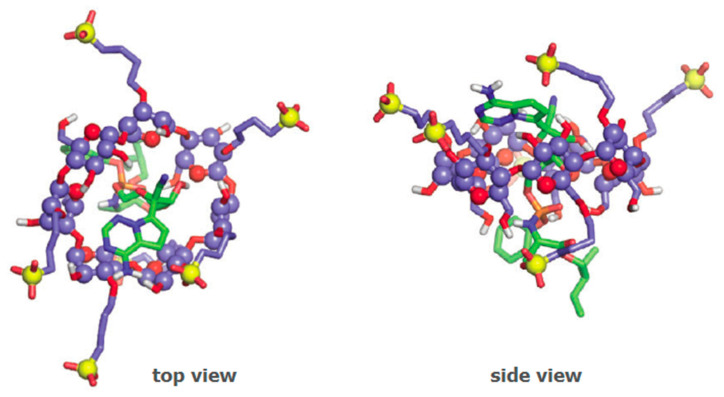
Structural representation of the interaction between remdesivir (molecules highlighted in green) and SBEβCD, as evaluated by molecular dynamics. Reproduced with permission from [86].

Remdesivir is a pro-drug with some hydrolytical instability, being degraded prior to absorption. For this reason, it requires intravenous administration (by perfusion). As a consequence of low aqueous solubility, an injectable formulation of remdesivir (200 mg dose) is only made possible when using SBEβCD (6 g per dose) as an excipient [85]. While SBEβCD only includes part of RSV (Figure 5), the excess amount ensures adequate solubilisation of the drug and makes this medicine the first example of a commercial antiviral formulation containing a cyclodextrin in its composition.

An overview of the herein described host-guest systems is presented in Table 3.

## 3. Cyclodextrins in Vaccines

### 3.1. Cyclodextrins as Vaccine Cryopreservatives: The Example of ad26.cov2.s

**Figure 6:**
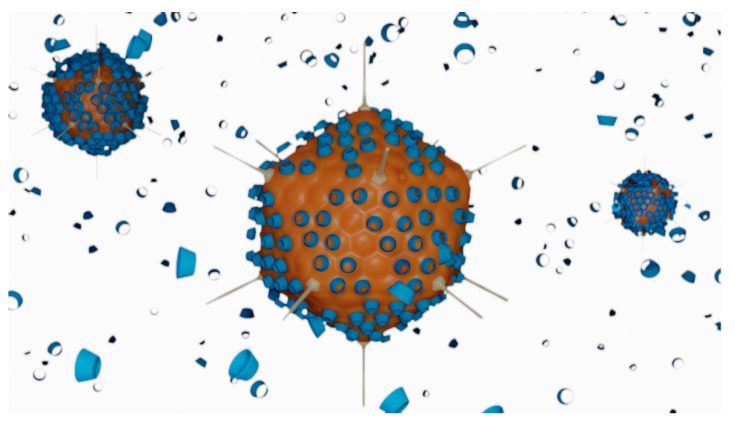
Schematic representation of the interactions between adenovirus particles (orange) and HPβCD molecules (blue) in the ad26.cov2.s vaccine. Cyclodextrin molecules are used in large excess and claimed to act as cryopreservatives, helping to stabilise the surface of the virus during the freeze-drying step of the vaccine preparation.

Several vaccines for SARS-CoV-2 are either under clinical development or already available under emergency use authorisation. The active ingredient in these vaccines is a nucleotidic chain to encode the immunogenic viral spike protein, with variations in the nature of the nucleic acids therein employed (mRNA or DNA) and in the kind of vector used to load the active component. The vaccine developed by Janssen with the name ad26.cov2.s has received emergency use authorisation of the FDA [87] and the EMA [88] in early March 2021. The vaccine, developed for the current SARS-CoV-2 pandemic, is based on viral DNA carried by a synthetic adenovirus vector and containing HPβCD as a cryopreservative (Figure 6) [89]. It is formulated as a re-suspendable powder for intramuscular administration, which implies that it requires a freeze-drying step during the preparation process. Low temperatures associated with the freeze-drying step are susceptible to causing damage to the surface of viruses. For this reason, HPβCD is included in the formulation as a cryopreservative, that is, to avoid cold-induced damage to the surface of the viral particles [90]. The process underlying the cryopreservative action of HPβCD with adenovirus particles is not elucidated. In semen cryopreservation, the preserving action was attributed to the interaction of HPβCD with cholesterol molecules on the membrane of these eukaryotic cells [91]. Adenoviruses, however, are not enveloped viruses, having only a protein capsid. The absence of the lipid envelope excludes the possibility of interaction with cholesterol or any other lipids.

### 3.2. Cyclodextrins as Vaccine Adjuvants

Cyclodextrins can help stimulate immune cell response, being thus suitable for usage as vaccine adjuvants. The first known cyclodextrin-adjuvanted commercial vaccine is a veterinary product with sulfolipo-β-cyclodextrin (SL-β-CD), a new derivative that was developed purposely to this end [92]. With an average substitution of 1.19 sulphate groups and 8.19 lipid residues per each cyclodextrin, SL-β-CD is an amphiphilic molecule that can be incorporated into oil/water emulsions. Squalene/water emulsions containing SL-β-CD were established as a new adjuvant medium for their toxicological safety and the ability to induce antibody response [92] and high lymphocyte proliferation in animal models [93]. We must note, however, that SL-β-CD is only approved for veterinary use.

In human vaccines, HPβCD is the best choice for adjuvanticity because it is already approved by the regulating entities and it has an excellent safety profile. Moreover, HPβCD is able to induce lymphocyte proliferation, especially the T-helper type 2 (Th2) cells. These cells, also known as CD4^+^ cells, are an important part of the immunisation effect of vaccines, contributing to maintaining a longer immune response. HPβCD is more advantageous than aluminium salts, which are currently the most commonly employed adjuvants. Unlike aluminium, HPβCD induces little immunoglobulin E (IgE) production, thus reducing the allergenic risk of the vaccine.

#### 3.2.1. Porcine Circovirus Vaccine

In 2013, the company Zoetis launched ‘Suvacyn PCV’, an inactivated recombinant porcine circovirus type 1 vaccine based on one of the viral proteins, which contained also the adjuvants squalene (32 mg/mL) and SL-β-CD (2 mg/mL) [94]. SL-β-CD was reported to induce a rapid onset of immunity and generate a better immune response as compared to Carbopol^®^ (polyacrylic acid), used in previous formulations of the vaccine.

#### 3.2.2. Human Influenza Vaccine

**Figure 7:**
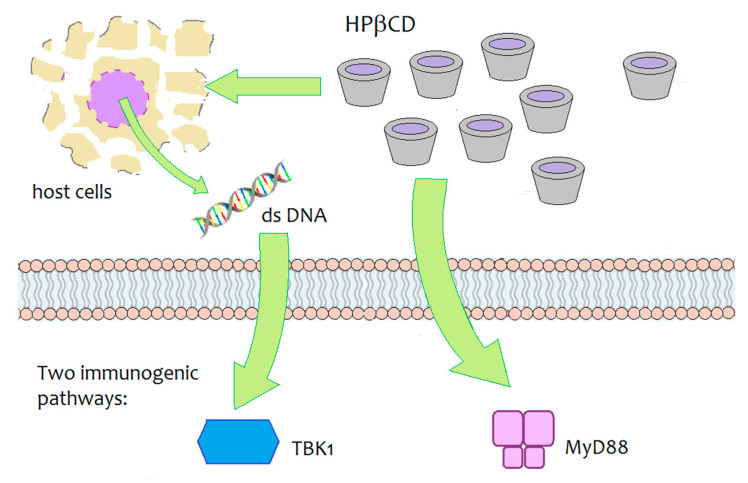
Schematic depiction of the biomolecular targets of HPβCD-mediated immunogenicity in mice [95].

Daiichi Sankyo, a Japanese pharmaceutical company, is developing an innovative vaccine for influenza that contains HPβCD as an innovative adjuvant agent. During the development studies of the vaccine with various animal models, HPβCD was shown to induce a synergic immune response by interacting with immunoglobulins, increasing antibody production by 30% and inducing the production of Th2 cells, responsible for long-term immune ‘memory’, as well as the generation of follicular B helper T cells (Thf) that in turn stimulate B cells (responsible for the long-term immune ‘memory’) [95,96]. The immunogenic activity of HPβCD, when co-administered with an antigen (ovalbumin), was investigated in mouse models. The underlying biomolecular mechanism was not fully elucidated, but results have demonstrated that two signalling pathways are involved— the MyD88-dependent pathway and the TBK1-dependent pathway (Figure 7) [95]. The TBK1-dependent signalling pathway was shown to be triggered by the release of dsDNA around the site of injection and it resulted in an enhanced Th2-type immune response. At the same time, HPβCD induced a MyD88-dependent response, which may help modulate the response upregulated by the dsDNA/TBK1 axis. The authors stated the need for further studies to identify the critical upstream/downstream pathways of MyD88 and TBK1 induced by local administration of HPβCD.

The positive results in animal studies encouraged the development of the human vaccine. The vaccine is designed for administration by the nasal route, reducing the discomfort of the yearly injections needed for the vaccination of elderly patients and those in risk groups. Currently, it is still under phase I trials [97]. Some results have already been published, showing that, in spite of the local administration at the nasal mucosa, the vaccine is able to generate a systemic immune response that occurs not only at the nasal mucosa but spreads to the entire organism to promote immunisation [98]. Approval of the first cyclodextrin-adjuvanted human vaccine will open the way for a new class of vaccines with higher safety profiles. The presence of aluminium salts in vaccines is a strong cause of concern and vaccination refusal for many patients. Replacing it with a safe adjuvant is expected to help regain the trust of patients and to contribute to widespread vaccination acceptance.

### 3.3. Cyclodextrins in mRNA Vaccines—A Future Trend?

Vaccines based on mRNA have set a new milestone in scientific development and in the control of the presently ongoing SARS-CoV2 pandemic. The development of these vaccines holds a new record for the shortest lab-to-market transition. This occurred because of the emergency use authorisation of regulated entities, which speeded the regulatory process. It also benefited from the strong advantages of the mRNA technology itself. Using nucleic acids as the active ingredient eliminates the time-consuming process of growing and inactivating viruses, which allowed the vaccines to be developed and tested as soon as the SARS-CoV2 viral genome was known. mRNA vaccines use the cells of the patient to produce the encoded viral proteins, generating immunostimulating particles inside the body. For this to happen, mRNA needs to be delivered to the cells while ensuring it does not suffer from the lytic action of the ribonucleases of the patient. The vector must also ensure that mRNA does enter the cytoplasm of the cells, where it will be free to exert its action. In the first two approved SARS-CoV2 mRNA vaccines, lipid nanoparticles were chosen as the vector owing to their good biocompatibility, facile preparation via microfluidics, and surface tuneability, which allows targeting specific cells [99]. These vaccines require, however, refrigeration at very low temperatures (one at −20 and the other at −70 °C), which may limit their widespread use [100].

**Figure 8:**
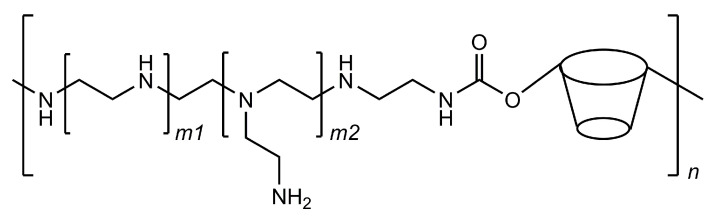
Structural representation of the cyclodextrin–polyethyleneimine conjugated polymer; the ratio of cyclodextrin residues in the polymer can be tuned by varying the length at the *m*1 and *m*2 subunits.

Following the release of the first two vaccines based on mRNA technology, researchers are seeking to further develop this technology by producing new vehicles for the nucleic acid chains. One of these strategies is based on cyclodextrin polymers that are designed to have polycationic charges so that they can interact with negatively charged molecules, such as DNA and RNA. Cyclodextrins help to stabilise the interactions, possibly by the inclusion of a few nucleobase residues and avoid the premature precipitation of the formed polycomplexes. More importantly, cyclodextrins help in making these systems more effective in the cell transfection phase because they are able to interfere with cellular membranes and increase their permeability. [101] These carriers are typically cyclodextrin–polyethyleneimine conjugated polymers (CD–PEI) that can be adapted to deliver different kinds of nucleic acid chains by customising the polyethyleneimine fragments (Figure 8).

A CD–PEI designed for mRNA vectorisation was recently reported [102]. CD–PEI and mRNA interacted to form adducts coined as nanocomplexes, which were shown to help mRNA reach the cytoplasm and transfect mouse dendritic cells (DC2.4 line) with high efficiency and to induce the expression of encoded proteins at high levels. The administration of the nanocomplexes was tested by different routes. Intramuscular injection-induced both IgG1 and IgG2a, with an IgG1 to IgG2a ratio of 1.34; intradermal administration primarily induced IgG2a antibodies and yielded an IgG1 to IgG2a ratio of 0.65. The authors noted that ratios between 0.5 and 2 indicate a mixed response of these two antibody classes.

While CD–PEIs remain as investigational compounds by lack of clinical studies to provide information on their biocompatibility, a similar class of cationic polymers (also based on iminium-β-CD units) was successfully used to deliver small interfering RNA to human cancer patients in a clinical trial [103]. These results may open the way for a range of future applications of CD-imine polymers in nucleic acid delivery.

## 4. Cyclodextrins as New Antiviral Agents

Cyclodextrins, well known for their role as excipients that help to solubilise, taste-mask, and stabilise active ingredients, are now gaining a new track in the role of medicinal agents. HPβCD, one of the most biocompatible and safe cyclodextrins, is already approved as an orphan drug for Niemann–Pick disease, a fatal neurodegenerative disorder [104,105], and other cyclodextrins are under evaluation for cardiovascular disease prevention and treatment due to their ability to form inclusion complexes with cholesterol in biological membranes [29] and reduce atherosclerosis [106,107]. The cholesterol-sequestering ability of cyclodextrins makes them interesting in other fields of medicine, namely, in antiviral applications, by disrupting the envelope of viruses that contain this biomolecule. In this regard and considering that the affinity to cholesterol follows the order β-CD ≈ RAMEB > HPβCD > TRIMEB [108], in addition to the solubilities of the various cyclodextrins, RAMEB has been the most extensively studied for viral inactivation. It must be stressed, however, that RAMEB is haemolytic and it has renal toxicity, and hence its use is limited to topical formulations. In turn, HPβCD can be used without restrictions. It was mainly studied against the HIV virus, having shown promising results in vitro but failing to ensure sustained protection in animal models that were subjected to repeated exposure to the virus (see Section 4.4). In the future, it would be meritorious to expand research on this molecule’s antiviral properties, determining its activity in vivo against a broad range of viruses. Moreover, more data on the immunomodulatory action of HPβCD should be gathered and correlated with antiviral activity results to search for possible synergies which may be very useful in the fight against viral infections.

### 4.1. Cyclodextrins against Influenza

The influenza virus has most of its membrane cholesterol organised in lipid-rich domains similar to the ‘lipid rafts’ of eukaryotic organisms. Indeed, most of the cholesterol in the viral membrane is taken from the cell membrane of the infected host upon exit of the viral particles. Cholesterol-rich domains are a good target of action for RAMEB, which is able to sequester this molecule and cause structural deformations in the viral membrane—literally poking holes in it, as observed by electron microscopy [109]. The result is a reduction in viral infectivity and viability [110,111]. Due to its strong action against influenza, a prospective application for RAMEB molecules would be in surface functionalisation of the fabric used in barrier methods (e.g., face masks) to render them more effective, by both adsorbing the viral particles and inactivating them.

Hemagglutinin A, one of the surface proteins of the influenza virus, is another therapeutic target for cyclodextrins. A new class of CDs, decorated with pentacyclic sterol analogues, was developed by grafting to the primary face of α-, β- and γ-CD scaffolds using ‘click’ chemistry reactions [112,113]. The antiviral activity of these new CDs was evaluated in vitro against influenza viruses grown inside canine epithelial kidney cells (MDCK line) to show that the derivatives of β-CD were the most potent ones, with IC_50_ values as low as 4.7 µM. Given that these cyclodextrins are new molecules, their safety also had to be evaluated. In vitro studies on non-infected MDCK cells showed that they are not toxic, but these datasets are still very preliminary. More in vitro tests on other cell lines, representative of various organs and tissues, and in vivo pre-clinical studies are lacking to help elucidate the clinical relevancy of these CDs to their full extent.

### 4.2. Cyclodextrins against the Dengue Virus

The dengue virus belongs to the Flaviviridae family, a taxon in which the Japanese encephalitis virus and the hepatitis C virus (see Section 4.3) are also included. Early results on membrane cholesterol sequestering by RAMEB to render these two kinds of viruses unable to enter their target cells and to replicate themselves were considered a very promising start, which would open the path towards a new anti-flaviviral therapeutic [114]. It is, however, tricky to use RAMEB to remove the cholesterol of these viruses after they have infected a patient; while in vitro assays have shown that RAMEB can deplete cholesterol of host immune cells, as demonstrated with a monocyte model (U937 myelomonocyte cell line) [115], and significantly reduces the infectivity rate of the dengue virus [116], it is known that cholesterol depletion is toxic to the patient, being responsible for the well-known haemolytical effect of RAMEB [33,34].

A more logical approach is to use RAMEB as an antiviral agent to interfere with the lifecycle of the dengue virus during the stage of the vectors—the Asian tiger mosquito (*Aedes albopictus*) and the common mosquito (*Aedes aegypti*). Proteomic studies have demonstrated that RAMEB strongly alters the protein metabolism of the virus inside the mosquito cells, especially with the lower expression of non-structural protein 1 (NS1), a protein required for viral replication and excretion [117]. Nevertheless, reducing the expression of this protein was insufficient to cause the reduction in the number of secreted viral particles in an in vitro culture of mosquito cells that were treated with RAMEB. Further investigation should thus be directed to a more comprehensive study of the effect of RAMEB on the mosquito stage of the virus life cycle. Due to the limitations of results obtained with the very simple models that are in vitro cell cultures, it is recommended to evaluate the activity of RAMEB on whole organisms, such as the mosquito larvae. This new methodology, if proven successful, will comprise a simple and eco-friendly method for the environmental control of dengue.

### 4.3. Cyclodextrins against Hepatitis C

Two different kinds of cyclodextrins have been evaluated against the hepatitis C virus: RAMEB and a family of fullerene-appended cyclodextrins. RAMEB was shown to hamper viral entry into macrophages [118] and to inhibit the assembly of viral particles inside hepatic carcinoma cells (Huh-7.5) [119]. Nevertheless, because of the aforementioned reasons, its systemic use is not recommended.

The fullerene-functionalised α-CDs were very promising inhibitors of viral internalisation into hepatic carcinoma cells (Huh-7), with IC_50_ values as low as 0.17 M [120]. Nothing is known, nevertheless, about their toxicological safety, which places them still very far from any prospect of clinical application.

### 4.4. HPβCD against HIV

HPβCD is able to remove some cholesterol molecules from the envelope of HIV and simian immunodeficiency virus (SIV), causing them disruptions that affect their infectivity [121,122]. Studies in mice show that this property of HPβCD allows it to block transmission of the virus by 91% when applied to the vagina: 10 out of the 11 treated mice did not get infected following HIV exposure [123]. Studies with rhesus macaques, the closest animal model to humans, have, however, yielded fewer promising results. The animals were treated with HPβCD by intravaginal administration and then subjected to SIV contact. HPβCD offered protection infection on the first contact with the virus, but not on the second inoculations (conducted 11 or 47 weeks later), which led to large-scale infection [124].

### 4.5. Labial Herpes Management with Cyclodextrins

Labial herpes is a recurrent condition in patients infected with the herpes simplex virus (HSV), which is triggered by stress and environmental changes. Usually controlled with resourcing to topical or systemic acyclovir, labial herpes may become challenging to handle in the case of acyclovir-resistant viral strains.

HPβCD was compared with polyethyleneglycol (PEG) for the prevention of labial herpes relapses in a double-blind, randomised trial with 40 patients [125]. During the six months of application of a moisturising gel with either HPβCD or PEG, both groups had fewer relapses than in the previous (non-treated) six months, in which each patient had up to 12 relapse episodes. The number of relapses with PEG was, interestingly, lower than that of the HPβCD group. This outcome was attributed to the higher moisturising ability of PEG and it led the authors to rule out HPβCD as a preventive agent for labial herpes episodes.

A new cyclodextrin, sulfoundecylthioether-βCD, was recently reported as active against both HSV-1 and the acyclovir-resistant HSV-2 strain. Time-dependant assays have further shown a significant reduction of viral titre after 5 min and the complete inactivation of the virus after 15 min, thus leading the authors to claim virucidal activity for this new cyclodextrin [126]. We highlight that the structure of sulfoundecylthioether-βCD bears a strong similarity with that of SBEβCD. The main difference is that it has a longer alkyl chain than SBEβCD and a thioether in the place of the ether. Given the excellent biocompatibility of SBEβCD, it can be expected that this new derivative will also be biocompatible.

## 5. Conclusions

This review presents a compilation of the different roles of cyclodextrins in the prevention and therapeutics of viral infections. While a large number of reports continue to study their classical role as solubilisers for hydrophobic drugs, this area is not very dynamic in terms of translation into the clinic. One likely reason is the high amount of cyclodextrins used in oral solid formulations, which increases the cost of the medicine. The golden exception is Veklury^TM^ (remdesivir), a recently approved drug for the treatment of SARS-CoV-2 infection which contains SBEβCD as the solubilising agent.

In our perspective, the field of vaccines is the one in which cyclodextrins have the strongest potential for development. The recently approved Janssen vaccine against SARS-CoV2 infection (ad26.cov2.s) sets a milestone for cyclodextrins in this field. Its global usage and the observation data resulting from monitoring the reports on any adverse effects from billions of patients will serve as a large-scale test to the safety of HPβCD, which it contains under the label of cryopreservative. If it passes this test, HPβCD will definitively consolidate its role as a compound of immense interest in biopharmaceuticals.

The main setback to a broader expansion of the usage of cyclodextrins in medicines and vaccines is the lack of information on their biological properties and, most importantly, on their safety. These studies are unavailable for the vast majority of chemically functionalised cyclodextrins. For the ones which have already been evaluated and approved for human use, such as HPβCD, reports continue to reveal more data on properties that were little explored, such as the effect on the immune system. It is currently known that HPβCD can act as an immunostimulant, with prolonged exposures (rats taking 0.4 g/kg/day for three months) causing increased monocyte (+150%) and overall white blood cells counts (15%) [127], and a single co-administration with an antigen (albumin) increasing proliferation of Th2 and Thf lymphocytes [95]. The molecular mechanisms of the immunostimulating action of HPβCD warrant, however, a more in-depth investigation. These studies should help understand the action of HPβCD in healthy individuals, immunocompromised patients, and those with autoimmune conditions, contributing to finding new therapeutic indications and possible counter-indications.

## Figures and Tables

**Table 1:** Summary of pharmaceutical products containing native and chemically modified cyclodextrins, their allowed daily intake from oral intake (ADI), restrictions to use (maximal dose), and main toxicity issues.

Cyclodextrin	Pharmaceutical Dosage Forms					ADI/Max Dose	Toxicity Concerns
	Oral	Nasal	Ocular	Dermal	Parenteral		
α-CD	—	—	—	—	✓	none	haemolytic
β-CD	✓	—	✓	✓	not allowed	5 mg/kg *per* day	nephrotoxic; haemolytic
γ-CD	✓	—	—	✓	—	none	haemolytic
HPβCD	✓	—	✓	✓	✓	n.s.	—
HPγCD	not allowed	✓	✓		not allowed	1.5% (*w*/*v*)	—
DIMEB	—	—	—	—	✓	n.s.	hepatotoxic
RAMEB	not allowed	✓	✓		not allowed	n.s.	nephrotoxic; haemolytic
SBEβCD	✓	—	—	—	✓	none	—

Notes: The check sign (✓) denotes known cases of dosage forms containing CDs for a particular delivery route; n.s. = not specified.

**Table 2:** In vitro inhibitory activity of ganciclovir and 10:1 associations of β-CD and γ-CD with ganciclovir on fibroblasts infected with various strains of human cytomegalovirus (HCMV).

Viral Strain	Ganciclovir	β-CD:GCV		γ-CD:GCV	
	IC_50_ (μM) ^1^	IC_50_ (μM)	Inc. Ratio ^2^	IC_50_ (μM)	Inc. Ratio
AD169	2.70 ± 0.55	0.20 ± 0.05	13.5	0.30 ± 0.01	9.0
RCL-1	14.50 ± 2.50	1.60 ± 0.12	9.1	—	—
1558	3.25 ± 0.62	0.20 ± 0.06	16.2	—	—
539	6.45 ± 0.82	2.50 ± 0.51	2.6	—	—
731	6.70 ± 0.55	5.80 ± 0.51	1.1	—	—
2288	18.25 ± 2.25	0.75 ± 0.80	24.3	—	—

^1^ Inhibitory activity determined by the ELISA method. Fibroblast MRC5 cells, in 96-well plates (4 × 10^4^ cells/well), were inoculated with HCMV suspension (m.o.i. = 0.1 PFU/cell) prior to the assay. Infected cells were treated by ganciclovir (GCV) or CD:GCV and the antiviral activity was measured after six days of incubation [51,52]. ^2^ ‘Inc. ratio’ expresses the activity increase ratio resulting from the presence of the cyclodextrin.

**Table 3:** Summary of CD adducts/inclusion complexes with antiviral drugs, showing the tested stoichiometries of inclusion, the apparent affinity constant (K*_app_*), and the advantages in their use.

Guest Drug	Host	H:G Ratio	K*_app_* (M^−1^) *^a^*	Benefits	Ref
Acyclovir	β-CD	1:1	—	↑ Solubility by c.a. 1.9-fold. ↑ Bioavailability by c.a. 1.2-fold	[40,41,43]
Acyclovir	β-CD	5:1	—	↑ Dissolution: 1.5 and 1.3 folds in HCl 0.1 N and PBS (pH 7.4)	[42]
Acyclovir	HPβCD	1:1	758 ± 7	Affords 100% drug dissolution in HCl 0.1 N ↑ Bioavailability by 1.6 fold regarding control (a 1:1 physical mixture of ACV and HPβCD)	[44]
Acyclovir	HPβCD	5:1	—	↑ Dissolution: 1.5 and 1.45 folds in HCl 0.1 N and PBS (pH 7.4)	[42]
Ganciclovir	β-CD	1:1	4976 *^b^*	(not tested)	[51]
Ganciclovir	β-CD	10:1	—	↑ In vitro antiviral potency Active against drug-resistant viral strains	[51,52]
Ganciclovir	γ-CD	10:1	—	↑ In vitro antiviral potency Active against drug-resistant viral strains	[51]
Ganciclovir dibutyrate diester	HPβCD	10:1	106.7 *^c^*	↑ In vitro corneal permeation by c.a. 2.6-fold (in a solution containing 5% HPβCD)	[53]
Efavirenz	β-CD	1:1	288 *^d^*	↑ Dissolution rate at 180 min by c.a. four-fold, that is, it was around 44%	[56]
Efavirenz	HPβCD	1:1	469 *^d^*	Dissolution rate increased to 60% at as early as 50 min and it remained at 60% until the end of the test (180 min)	[56]
Efavirenz	RAMEB	1:1	1073 *^d^*	↑ Dissolution rate at 180 min by c.a. six-fold, that is, it was around 60%	[56]
Efavirenz	γ-CD	3:2	—	↑ Solubility	[54,58]
Rilpivirine	β-CD	2:1	—	↑ Dissolution in acidic medium	[63]
Saquinavir	β-CD	—	4086	↑ Dissolution rate at 60 min by c.a. two-fold	[69,70]
Saquinavir	RAMEB	3:1 *^e^*	6148 *^f^*	100% dissolution rate	[67]
Saquinavir mesylate	RAMEB	3:2 *^e^*	—	100% dissolution rate ↑ Oral bioavailability: earlier *t*_max_ and C_max_ raised 10-fold	[67]
Lopinavir	HPβCD	1:1	443.9	↑ Solubility 50% dissolution at 120 min from the kneaded product	[73]
Lopinavir	RAMEB	1:1	582.9	↑ Solubility 30% dissolution at 120 min from the kneaded product	[73]
Lopinavir	γ-CD	1:1	305.0	↑ Solubility 55% dissolution at 120 min from the kneaded product 7% dissolution at 60 min from the spray-dried (SD) product	[73,77]
Lopinavir	HPγCD	1:1	—	↑ Solubility 14% dissolution at 60 min from the SD product	[77]
Lopinavir	(HP)_17_γCD	1:1	—	↑ Solubility 33% dissolution at 60 min from the SD product	[77]
Oseltamivir	β-CD	1:1	—	Taste-masking effect	[79]
Remdesivir	SBEβCD	14:1 *^g^*	—	Enables the preparation of an injectable formulation	[85]

*^a^* K*_app_* values are presented for the 1:1 complex and given, when available, as mean ± standard deviation. *^b^* K*_app_* was determined from the solubility isotherm data using UV–Vis quantification at 253 nm. *^c^* K*_app_* was determined from the solubility isotherm data using HPLC quantification. *^d^* K*_app_* was determined from the solubility isotherm data using UV–Vis quantification at 246 nm. *^e^* Calculated from the reported 11.56 wt% of saquinavir base and 27.6 wt% of saquinavir mesylate in the solid inclusion compounds. *^f^* K*_app_* was determined from the solubility isotherm data measured in phosphate buffer at pH 7.4. *^g^* Calculated from the amounts of remdesivir (200 mg) and SBEβCD (6 g) available in the commercial formulation.

## Abbreviations

ACV	acyclovir
API	active pharmaceutical ingredient
BCS	biopharmaceutical classification system
CD	cyclodextrin
CHMP	committee for medicinal products for human use
DIMEB	heptakis-2,6-di-O-methyl-β-CD
DMEM	Dulbecco’s modified eagle medium
DSC	differential scanning calorimetry
FDA	US Food and Drugs Administration
EFV	efavirenz
ELISA	enzyme-linked immunosorbent assay
EMA	European Medicines Agency
EMEM	Eagle’s minimum essential medium
GI	gastrointestinal
GCV	ganciclovir
HCMV	human cytomegalovirus
HCV	hepatitis C virus
HPβCD	(2-hydroxyl)propyl-β-CD
HIV	human immunodeficiency virus
HSV	herpex simplex virus
IgG	immunoglobulin G
LPV	lopinavir
MERS	Middle Eastern respiratory syndrome
MLK	montelukast sodium
m.o.i.	multiplicity of infection
MV	measles virus
MyD88	myeloid differentiation primary response protein 88
NS-1	non-structural protein 1
OSV	oseltamivir phosphate (Tamiflu^TM^)
PAA	poly(amidoamine)
PBS	phosphate buffer saline
PEG	polyethyleneglycol
PEI	polyethyleneimine
RBV	ribavirin
RAMEB	randomly methylated β-CD
RPV	rilpivirine
RSV	remdesivir
RTV	ritonavir
SARS	severe acute respiratory syndrome
SBEβCD	sulfobutylether-β-cyclodextrin (Captisol^®^)
SD	spray-dried
SL-β-CD	sulfolipo-β-CD
SLS	sodium lauryl sulphate
SQV	saquinavir
TBK1	TANK-binding kinase 1 Th2, type 2 T-helper
TRIMEB	heptakis-2,3,6-*tris*-O-methyl β-CD
VZV	varicella-zoster virus
