# Correction: Braga et al. Cyclodextrins in Antiviral Therapeutics and Vaccines. *Pharmaceutics* 2021, *13*, 409

**DOI:** 10.3390/pharmaceutics14030499

**Published:** 2022-02-24

**Authors:** Susana Santos Braga, Jéssica S. Barbosa, Nádia E. Santos, Firas El-Saleh, Filipe A. Almeida Paz

**Affiliations:** 1 LAQV-REQUIMTE, Department of Chemistry, University of Aveiro, 3810-193 Aveiro, Portugal; jessicambarbosa@ua.pt (J.S.B.); nadiaasantos@ua.pt (N.E.S.); 2 CICECO—Aveiro Institute of Materials, Department of Chemistry, University of Aveiro, 3810-193 Aveiro, Portugal; filipe.paz@ua.pt; 3 Ashland Specialty Ingredients, Paul-Thomas Strasse, 56, D-40599 Düsseldorf, Germany; FElSaleh@ashland.com

## Error in Figure

In the original publication [1], there was a mistake in **Figure 2** as published. **The structures of hydroxypropyl-β-cyclodextrin and sulphobutyl-β-cyclodextrin were not representing the most chemically abundant species that are formed during the reactions of chemical substitution of OH groups by 2-hydroxypropyl and sulfobutyl residues, respectively.** The corrected **Figure 2** appears below this text.

**Figure 2:**
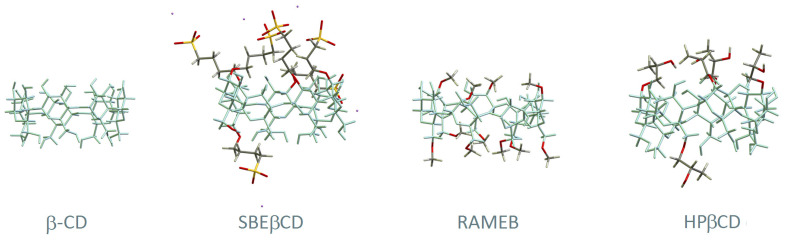
Structural representation of β-CD and three of its derivatives: (2-hydroxy)propyl-beta-cyclodextrin (HPβCD), randomly methylated beta-cyclodextrin (RAMEB), and sulfobutyl ether β-CD (SBEβCD). The main skeleton of β-CD is represented in blue and the substituent groups are highlighted with different colours (carbon in grey, oxygen in red, hydrogen in white, sulphur in yellow and sodium in purple).

The authors apologise for any inconvenience caused and state that the scientific conclusions are unaffected. The original publication has also been updated.
